# HSV-pneumonitis in a patient with lung cancer receiving check point inhibitors – a case report

**DOI:** 10.1186/s41479-020-00079-y

**Published:** 2021-01-25

**Authors:** Johannes Sumer, Frederike Waldeck, Nadja Fischer, Christina Appenzeller, Markus Koster, Martin Früh, Werner C. Albrich

**Affiliations:** 1 Division of Infectious Diseases and Hospital Epidemiology, Cantonal Hospital St. Gallen, Rorschacher Strasse 95, 9007 St. Gallen, Switzerland; 2 Division of Pathology, Cantonal Hospital St. Gallen, Rorschacher Strasse 95, 9007 St. Gallen, Switzerland; 3 Division of Oncology, Cantonal Hospital St. Gallen, Rorschacher Strasse 95, 9007 St. Gallen, Switzerland; 4 University of Bern, Bern, Switzerland; 5 Division of Internal Medicine, Cantonal Hospital St. Gallen, Rorschacher Strasse 95, 9007 St. Gallen, Switzerland

**Keywords:** Herpes simplex virus, Pneumonitis, Case report, Immune check point inhibitors

## Abstract

**Background:**

Herpes simplex virus (HSV) is commonly associated with oro-facial and genital manifestations. It rarely causes encephalitis and even less commonly, in heavily immunosuppressed patients, visceral disease or bronchopneumonitis. We present a case of cytologically-proven, PCR-positive HSV-1 tracheobronchitis and pneumonitis in a patient with less severe immunocompromise.

**Case presentation:**

A 64 year old white man with steroid-induced diabetes mellitus and progressive small-cell bronchial carcinoma despite chemo- and immunotherapy with two checkpoint inhibitors presented with symptoms of lower respiratory tract infection. Community-acquired pneumonia was suspected and empirical broad-spectrum antibacterial treatment was initiated. Chest CT-scan revealed ground-glass opacities and tree-in bud lesions. Cytology of BAL showed extensive cytopathic effects typically caused by infection with herpes virus and PCR confirmation of HSV-1. Acute phase HSV serology was positive for IgG and borderline for IgM. The patient deteriorated clinically due to tumor progress and infection despite high-dose acyclovir therapy and died 2 weeks after admission.

**Conclusions:**

We report an unusual case of fatal HSV-1 pneumonitis due to reactivation in a patient with lung cancer, steroid-induced diabetes and treatment with two checkpoint inhibitors. In immunosuppressed patients with non-improving pneumonia invasive diagnostic procedures are warranted including cytology and molecular diagnostics.

## Background

Primary infection with Herpes simplex virus 1 (HSV-1) or 2 (HSV-2) occurs after viral inoculation of mucous membranes or skin. Clinical presentation ranges from asymptomatic courses to symptoms of fever, general malaise and local vesicular rash or potentially fatal organ manifestations [1].

After primary infection the virus remains in a latent stage forming a reservoir in nerve cell bodies. Reactivation can be triggered by physical or emotional stress [2–5], trauma to the site of primary infection, fever and immunosuppression among other causes. Recurrence usually affects the site of primary infection, but may spread to adjacent areas via peripheral nerves or epithelial cells [6, 7].

While rarely seen in immunocompetent adults [8], HSV-1 infection of the lower respiratory tract (LRT) has been described in immunosuppressed and critically-ill patients, where HSV-1 is frequently detected in LRT samples either due to sample contamination from oropharyngeal sites of reactivation or direct extension from the upper airways leading to tracheobronchitis [9].

There is little clinical information whether checkpoint inhibitors have an impact on frequency of HSV-infections, though there is some evidence that these immunomodulatory drugs may even be advantageous in clearance of HSV-1 infections (20). Here, we report a case with proven HSV-pneumonitis in a patient with double checkpoint blockage and review the existing literature. To the best of our knowledge this is the first report describing HSV-pneumonitis in a patient receiving corticosteroids and checkpoint inhibitors.

## Case presentation

A 64 year old white male patient was admitted to our tertiary care hospital with dyspnoea, cough and malaise. Past medical history was significant for small-cell bronchial carcinoma (diagnosed 11 months prior) with lymph node, cerebral and adrenal metastases. He was treated with chemotherapy (cisplatin/etoposide) and radiotherapy due to cerebral and mediastinal progress. Second line immunotherapy was established with double checkpoint inhibitor therapy with ipilimumab/nivolumab and additionally dexamethasone (12 mg/d, started 1 month ago after seizure) because of cerebral edema as well as for immune-related thrombocytopenia under therapy with checkpoint inhibitors. There was a history of steroid-induced diabetes mellitus and subsegmental pulmonary emboli 10 months prior to presentation.

On clinical examination - after two cycles nivolumab/ipilimumab and still under dexamethasone - the patient was afebrile, hypotensive and normocardic. Pulmonary exam revealed reduced breath sounds bilaterally, poor oxygenation (oxygen saturation 91% with 2 L O2) and a respiratory rate of 19/min.

**Fig. 1:**
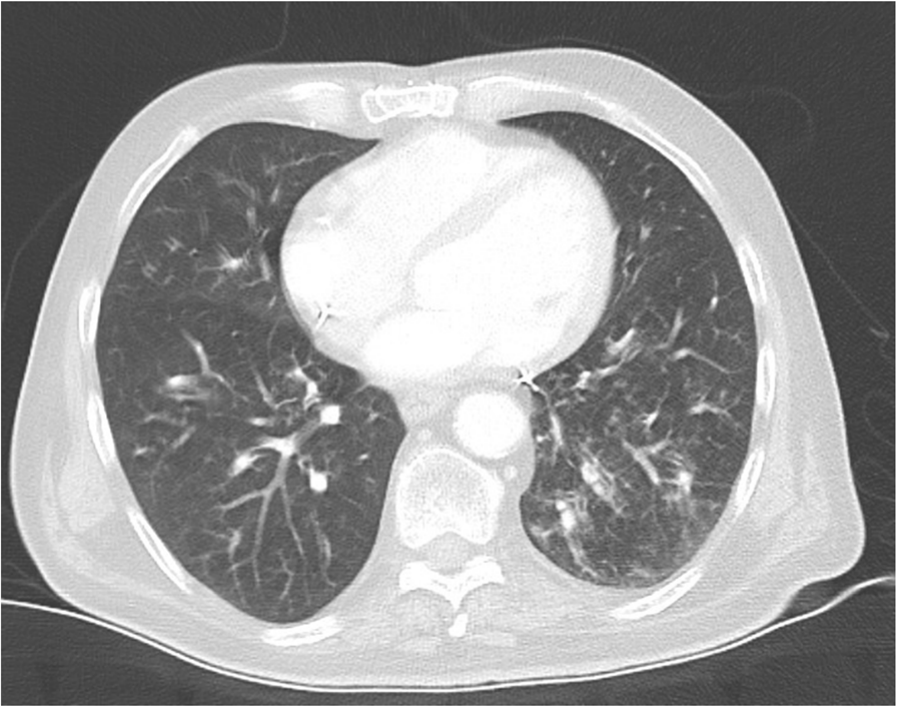
Axial window CT-scan on day 1 showing pneumonitis with tree-in bud pattern and ground glass opacities

**Fig. 2:**
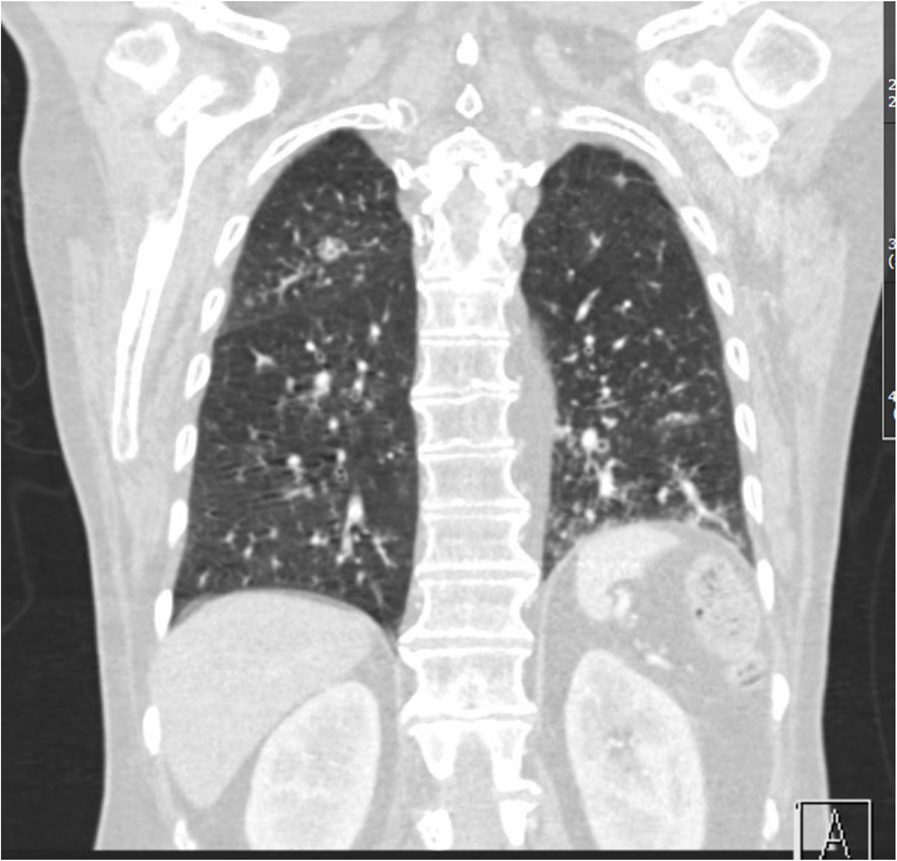
Coronal window CT-scan on day 1 showing pneumonitis with tree-in bud pattern and ground glass opacities

Laboratory examinations on admission revealed a markedly elevated CRP (424 mg/l) and LDH (520 U/l), slightly elevated alanine transaminase (61 U/l), exacerbated pancytopenia with anaemia (Hb 120 g/l), thrombocytopenia (82G/L) and leukocytopenia (2.1G/L) with normal granulocytes (absolute 1.3G/l) and lymphopenia (5%, absolute 0.1G/l). A chest CT-scan showed signs of pneumonitis with new tree-in bud pattern and peribronchially localized ground glass opacities (Figs. 1 and 2). The bronchial mass was stable compared with a CT performed 3 weeks ago. Legionella antigen in urine was negative.

**Fig. 3:**
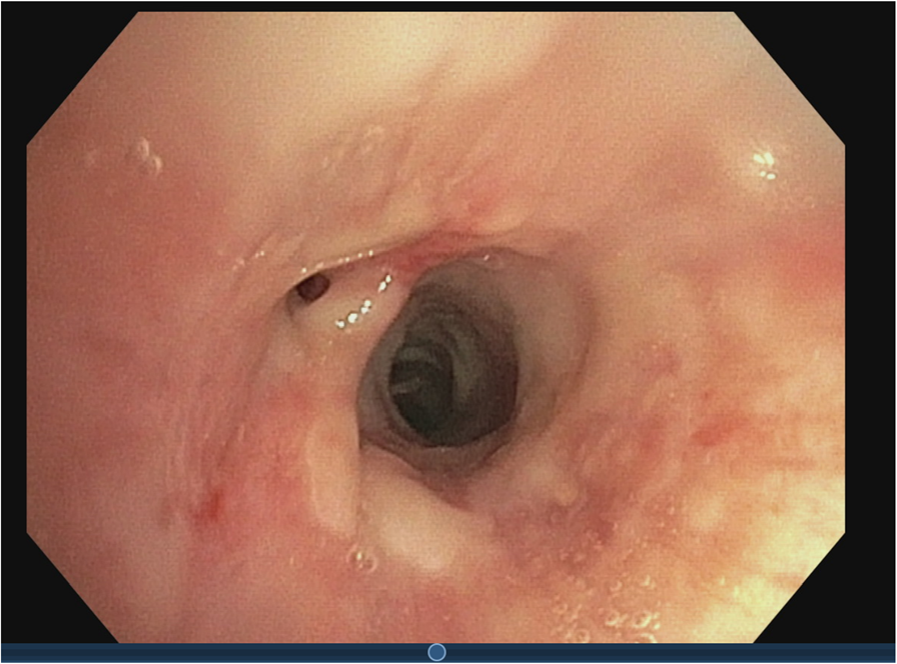
Bronchoscopy shows reddened mucosa with little white mucus (view in lower lobe left)

There was no clinical improvement despite improving signs of inflammation with antibiotic treatment with amoxicillin/clavulanic acid and clarithromycin. Therefore bronchoscopy, which showed macroscopic erythematous lesions in the trachea, and bronchoalveolar lavage (BAL) were performed (Fig. 3). Microscopically a lot of alveolar macrophages and some polymorphonuclear leukocytes (PMN) were visible with normal respiratory flora, so BAL was perfectly consistent with a good sample of the lower airways with an acute inflammation. There was no bacterial or mycobacterial growth on routine culture. A viral respiratory multiplex polymerase chain reaction (PCR, AllplexTM Respiratory Panel 1–3 [Seegene)]) and antigen for *Pneumocystis jirovecii* were negative. Cytology revealed elevated cell count (833 × 10^6^/L cells) and neutrophilic granulocytes (68.7%).

**Fig. 4:**
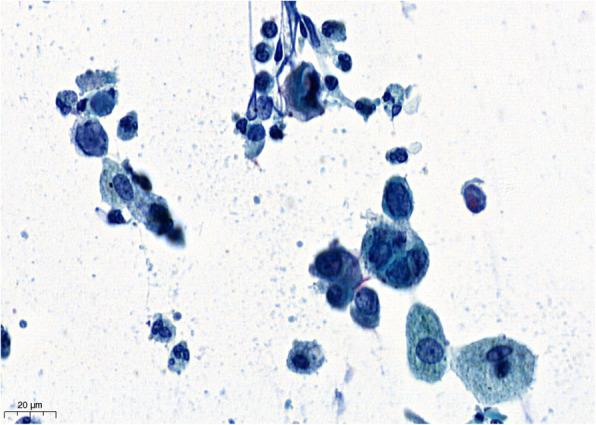
Cytology of BAL fluid showing cytopathic effect

**Fig. 5:**
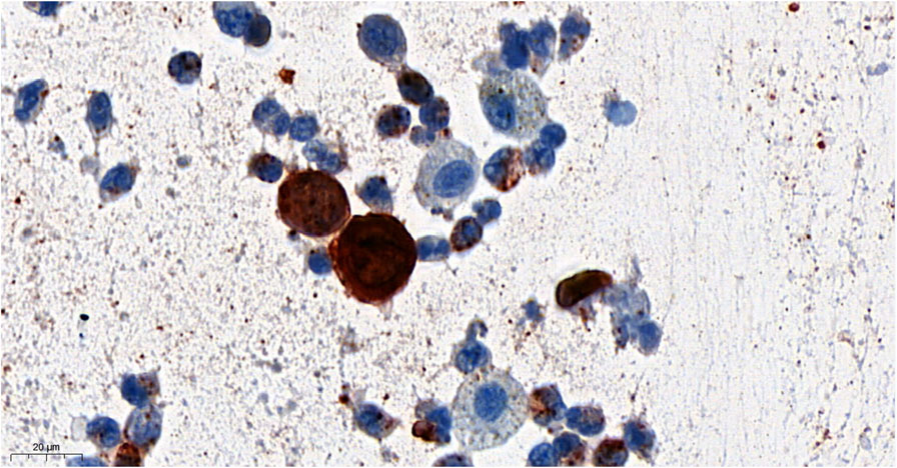
Immunohistochemistry of BAL, showing immuncytochemic positivity in viral loaded cells. The antibody (Diagnostic Biosystems) reacts with HSV type I and II specific antigens. It reacts with all the major glycoproteins present in the viral envelope as well as with at least one core protein

There was an extensive cytopathic effect (Fig. 4) including a positive immunocytochemical stain for HSV-1/2 suggesting HSV-infection in numerous cells (Fig. 5). Fungal or malignant cells were not detected.

Retesting of bronchial secretions with non-quantitative HSV-PCR (Artus® HSV-1/2 QS-RGQ Kit (QIAgen)) detected HSV-1 (Ct value 18.97). Serology (Liaison®HSV-1/2 IgG, Liaison®HSV-1/2 IgM, DiaSorin) showed positive HSV 1/2 IgG (OD-index: 29.0; cut-off: 1.0) and borderline positive (OD-index: 0.97; cut-off: 1.1) IgM. Antiviral therapy with acyclovir 10 mg/kg body weight was started intravenously upon receipt of cytology 3 days after admission. The dose was transiently decreased to 5 mg/kg body weight for 1 day, but in light of reduced vigilance suggesting a possible affection of the central nervous system the dose was increased again to 10 mg/kg body weight on the following day.

Despite improvement of inflammatory parameters the patient continued to deteriorate clinically. With progressive cognitive deterioration cerebral HSV involvement was suspected but was not proven as a diagnostic lumbar puncture could not be performed due to severe and progressive thrombocytopenia (minimum 22G/L) which was attributed to the immunotherapy. A cranial MRI eventually showed progressive cerebral metastases as likely cause of deterioration.

Because of the significantly progressive underlying disease management was altered to palliative care after 10 days of acyclovir. The patient died 2 weeks after admission. No autopsy was performed.

## Discussion

We report a fatal case of cytologically and PCR proven HSV-1 tracheobronchitis and pneumonitis in a solid organ cancer patient with lymphopenia, steroid-induced diabetes and treatment with dexamethasone and checkpoint inhibitor.

Diagnosis of HSV pneumonia may be challenging due to lack of typical clinical and radiologic features [10]. Given very high seroprevalence worldwide, serology is not useful in most cases except when high IgM titers are present suggesting primary infection, though IgM blips are described during the chronic phase of HSV in apparently health persons [11]. In our case borderline positive IgM and high titers of IgG suggested reactivation and made primary infection unlikely.

Detection of HSV deoxyribonucleic acid by PCR may be only of limited diagnostic utility, since discrimination between relevant infection and simple viral shedding is difficult. However, the low Ct value in our case reflected a high viral load and confirmed a severe and clinically relevant infection. Our patient had as well tracheobronchitis, evident during bronchoscopy with typical erythematous lesions, as groundglass opacities consistent with HSV pneumonitis.

Proof of HSV infection can be provided by histopathological or cytological examination of infected tissue showing a typical cytopathic effect [12]. As in our case cytological workup showed unequivocal confirmation of HSV-infection and immunocytochemical staining further confirmed diagnosis.

Cytological features suggesting HSV infection in BAL samples have only rarely been described in routinely processed samples [13]. A diagnosis of HSV is suggested based on a number of well described and characteristic cellular changes. Typical signs of infected cells are multinucleated, swollen nuclei clustered tightly together leading to a characteristic molding of nuclear contours. The presence of viral inclusions leads to a loss of chromatin pattern and the nuclei are observed to take on an empty homogenized or ‘ground glass’ appearance with a prominent nuclear membrane, also known as Cowdry type B nuclei [14, 15]. Strongly eosinophilic inclusions develop in the nucleus known as Cowdry type A inclusions, which are often wedge-shaped or triangular and can appear refractile later during the infective process Cowdry bodies are characteristic but non-specific for HSV [12, 16]. The typical findings in cytology and positive PCR confirmed HSV-1 infection in our patient with the clinical diagnoses of tracheobronchitis and pneumonitis.

Whether HSV was the single causative pathogen leading to the initial respiratory decline or if it occurred as a superinfection of an unidentified bacterial cause is difficult to judge retrospectively since inflammatory parameters decreased during empirical antibiotic therapy in contrast to his clinical condition.

HSV commonly infects patients with impaired cellular immunity, the incidence of pulmonary involvement lies between 36 and 65% according to Aisenberg et al. [17] and Ramsey et al. [7]. Witt et al. screened a total of 2480 patients with rheumatoid arthritis, vasculitis and SLE retrospectively for pneumonia. Among those there were 63 episodes of hospital admission with respiratory deterioration, that were ultimately diagnosed as pneumonia or pneumonitis. 6 cases (10% of admissions for pneumonia) were identified to be associated with HSV-1 in lower respiratory tract, but HSV was suspected to play a causative role only in 2 episodes. Detection of HSV-1 was associated with stronger immunosuppressive regimens and vasculitis [18].

Our patient’s risk was likely related to dexamethasone (12 mg/d), which was started about 1 month before hospitalization and which is known to predispose to infections with Herpesviridae [19]. In addition, lymphopenia is a strong risk fact for infection with herpes viruses. In a cohort study of patients with solid tumors and lower respiratory tract HSV infection Aisenberg et al. described lymphopenia as common and severe lymphopenia was present in nearly half of the entire cohort [17]. Lymphopenic patients are also at risk for Varicella zoster virus, Epstein Barr Virus, Cytomegalovirus and Human Herpes virus 6 infections [20–22]. Our patient had severe lymphopenia which also put him at increased risk for HSV lower respiratory tract infection as shown previously [17].

Bacterial infections like pneumonia, intra-abdominal infections, but also varicella zoster virus infection, pulmonary aspergillosis and pneumocystis pneumonia have been described in cancer patients receiving immune checkpoint inhibitors [23]. There is no intrinsically increased risk of infection – especially VZV or HSV-infections - regarding ipilimumab and nivolumab, but an increased risk seems to be associated with additional immunosuppressive treatments or conditions, particularly corticosteroids [23]. There is even data showing that immune checkpoint inhibitors may reverse the functional exhaustion status of virus-specific T cells allowing to mount appropriate T cell responses and virus clearance [24]. In fact, Jeon et al. demonstrated that local PD-L1 blockade in the cornea resulted in enhanced HSV-1 clearance [25]. We did not find reports of severe HSV-infections in patients receiving immune checkpoint inhibitors or evidence for an increased risk. However, Foukas et al. reported a case of severe interstitial pneumonitis with concomitant detection of HHV-6 in a patient under nivolumab [26].

There is equivocal data whether immunosuppression leads to higher mortality in patients with HSV pneumonitis especially those receiving immune checkpoint inhibitors. Interestingly, in an older study of 42 patients with respiratory cultures positive for HSV, immunocompetent patients had a more severe presentation with worse bronchospasm, greater difficulty weaning and worse outcome than immunocompromised patient [27]. Mortality rates of patients with non-solid organ tumors and HSV-isolation from the LRT have been reported as high as 27% [28]. In a series of 45 patients with proven, probable or possible HSV pneumonia, 38% suffered from pulmonary malignancy and 22% died [17].

Since our patient was very acutely ill and immunosuppressed with lymphopenia, steroids and checkpoint inhibitors we treated him with acyclovir, though no guidelines exist regarding treatment indications of HSV-pneumonitis. Simoons-smit et al. recommended starting therapy when HSV is isolated from respiratory tract only if it occurs in immunocompromised patients (e.g. patients with malignancies), if there is evidence of pulmonary parenchymal invasion or unexplained clinical deterioration [29]. Our patient fulfilled all three criteria.

Generally there is conflicting data on the benefit from antiviral therapy in HSV pneumonitis [29]. In a small retrospective cohort study by Camps et al. acyclovir treatment showed no effect on overall survival in 28 patients with respiratory specimens positive for HSV [30]. A recent study by Luyt et al. similarly showed no benefit of preemptive use of acyclovir in mechanically ventilated patients with HSV oropharyngeal reactivation in terms of the number of ventilator-free days but improved 60 day survival [31]. In contrast, in a retrospective study acyclovir therapy resulted in lower mortality and all 6 patients with HSV-pneumonitis who received acyclovir survived [17].

In conclusion, we report the first fatal case of HSV-1 pneumonitis in a lymphopenic solid organ cancer patient receiving corticosteroids and checkpoint inhibitors. While we do not know what the relative contributions of all these predisposing factors were to the fatal course, it makes this presentation a relevant warning for clinicians taking care of patients with other comorbidities or adjunct immunosuppressive therapies. Despite the notion that checkpoint inhibitors themselves do not increase the risk of HSV-infection, checkpoint inhibitors, particularly combinations as in our case, may often cause immune mediated pneumonitis. Its radiological pattern frequently shows interstitial changes, though there are no pathognomonic findings [24]. In such situations adjunctive treatment with steroids is established, which may be harmful in case of undetected HSV infection. Therefore meticulous diagnostic assessment is warranted, especially in patients with lymphopenia and recent steroid treatment in order to avoid missing alternative diagnoses.

Moreover this case illustrates the importance of obtaining cytological examination in the diagnostic work-up of suspected pulmonary infections, particularly in immunocompromised hosts with refractory pneumonia where it should be combined with broad molecular diagnostics. Further studies are needed to define optimal antiviral treatment and treatment duration as well as to clarify the potential impact of checkpoint inhibitors on risk of herpes and other viral infections.

## Data Availability

All patient data that support this case report are included in anonymized form in the published article.

## Abbreviations

HSV: Herpes simplex virus
BAL: Bronchoalveolar lavage
LRT: Lower respiratory tract
PD1: Programmed cell death protein 1
LDH: Lactate dehydrogenase
CRP: C-reactive protein
HB: Hemoglobin
CT: Computer tomography
PCR: Polymerase chain reaction
